# Growth of vertically aligned ZnO nanorods using textured ZnO films

**DOI:** 10.1186/1556-276X-6-524

**Published:** 2011-09-07

**Authors:** Francisco Solís-Pomar, Eduardo Martínez, Manuel F Meléndrez, Eduardo Pérez-Tijerina

**Affiliations:** 1 Centro de Innovación, Investigación y Desarrollo en Ingeniería y Tecnología de la UANL-PIIT, Apodaca, Nuevo León 66600, México; 2 Facultad de Ciencias Físico-Matemáticas, Universidad Autónoma de Nuevo León, San Nicolás de los Garza, Nuevo León 66451, México; 3 Centro de Investigación en Materiales Avanzados S. C., Unidad Monterrey-PIIT, Apodaca, Nuevo León 66600, México; 4 Department of Materials Engineering, (DIMAT), Faculty of Engineering, University of Concepción, 270 Edmundo Larenas, Casilla 160-C, Concepción, Chile

**Keywords:** vertical-aligned ZnO nanorods, atomic layer deposition, hydrothermal method

## Abstract

A hydrothermal method to grow vertical-aligned ZnO nanorod arrays on ZnO films obtained by atomic layer deposition (ALD) is presented. The growth of ZnO nanorods is studied as function of the crystallographic orientation of the ZnO films deposited on silicon (100) substrates. Different thicknesses of ZnO films around 40 to 180 nm were obtained and characterized before carrying out the growth process by hydrothermal methods. A textured ZnO layer with preferential direction in the normal *c*-axes is formed on substrates by the decomposition of diethylzinc to provide nucleation sites for vertical nanorod growth. Crystallographic orientation of the ZnO nanorods and ZnO-ALD films was determined by X-ray diffraction analysis. Composition, morphologies, length, size, and diameter of the nanorods were studied using a scanning electron microscope and energy dispersed x-ray spectroscopy analyses. In this work, it is demonstrated that crystallinity of the ZnO-ALD films plays an important role in the vertical-aligned ZnO nanorod growth. The nanorod arrays synthesized in solution had a diameter, length, density, and orientation desirable for a potential application as photosensitive materials in the manufacture of semiconductor-polymer solar cells.

**PACS:**

61.46.Hk, Nanocrystals; 61.46.Km, Structure of nanowires and nanorods; 81.07.Gf, Nanowires; 81.15.Gh, Chemical vapor deposition (including plasma-enhanced CVD, MOCVD, ALD, etc.)

## Background

ZnO wurtzite hexagonal phase is one of the most important functional materials due to its excellent physicochemical properties and its diversity in terms of morphologies, properties, and applications [1,2]. The excellent properties of ZnO include direct band gap (3.37 eV) and high optical gain of 300 cm^-1 ^(100 cm^-1 ^for GaN) at room temperature, large saturation velocity (3.2 × 10^7 ^cm/s), high breakdown voltage, and large exciton binding energy (60 meV). These versatile properties of ZnO provide an opportunity to recognize it as one of the most multifunctional materials; therefore it can be used for ultraviolet lasers, light-emitting diodes, photo-detectors, piezoelectric transducers and actuators, hydrogen storage, chemical or biosensors, surface acoustic-wave guides, solar cells, and photo catalysts, among others [2-4]. As mentioned, one of the qualities of this material is that it can be obtained in different types of nanostructures, being 1D ZnO nanostructures such as nanorods and nanowires the most used owing to their great prospects in fundamental physical science, nanotechnology applications, nano-optoelectronics, and photovoltaic devices. Hence, it is desirable to develop fast, simple, and mild routes for the synthesis of 1D high crystalline quality ZnO nanostructures in a large area, to explore their diverse applications. Among various applications of this material, one can say that the utilization of ZnO nanostructures as photo-electrodes in dye-sensitized solar cells (DSSCs) has received considerable attention currently due to their compatibility with the commonly used TiO_2 _materials [5-8]. Besides, ZnO shows higher electronic mobility and similar energy level of the conduction band than TiO_2 _which makes ZnO a candidate to be used as a photo-electrode material for the fabrication of efficient DSSCs. Several methods have been used to grow nanowires and nanorods such as: vapor-liquid-solid (VLS) [3,4], metal organic vapor-phase epitaxy (MOVPE) [9], pulsed laser deposition (PLD) [5,6] solution, and hydrothermal methods [7,8]. In some cases, these arrays were synthesized at temperatures ranging from 400°C to 600°C through metal-organic chemical vapor deposition (MOCVD) [10-12], PLD [13], and chemical vapor transport (CVT) [14] implying high temperature, complex, and expensive processes. In addition, high-quality vertical ZnO nanowire arrays have been grown using both (1) heteroepitaxy with Al_2_O_3 _or single-crystalline GaN, which is currently limited to expensive substrates [15-17] and (2) homoepitaxy with a textured ZnO thin film that is deposited on top of a non-epitaxial substrate which act as a nanorod nucleation layer [18-22]. Neither approach is particularly low-cost, versatile, or promising for the fabrication of high-performance ZnO nanowire optoelectronic devices, including solar cells.

With the aim to explain the nanorod alignment, Zhang et al. hypothesized that a textured ZnO wetting layer formed prior to nanorod growth favors the alignment [22]. If this notion is correct, it should be possible to control the crystallography of the seed layer film to obtain nanorods by an alternative method like the hydrothermal treatment and thus enhance the process to achieve aligned nanorods. Therefore, one could then create surfaces that would work as growth seeds for the ZnO nanowires on an assortment of substrates using any nanowire growth technique, e.g., gas-phase or solution phase. In this work, vertically aligned ZnO nanorods on Si(100) substrates were synthesized using a hybrid atomic layer deposition (ALD) and hydrothermal method. For accomplishing this, ZnO films were prepared by ALD at different thicknesses to obtain seed layers of different crystallographic nature.

The purpose of this work is to study the effect of crystalline orientation of the seed layer on the ZnO nanorods growing by hydrothermal. In this way the aim is to determine the best conditions to grow perfectly aligned and uniform ZnO nanorods and provide the foundation to achieve a better controlled and large-scale synthesis of ZnO nanorods.

## Experimental

The fabrication procedure for the growth of the nanorods consists of two steps: (1) preparation of a seed-textured ZnO thin layer by ALD and (2) the nanorod array growth by hydrothermal.

### Synthesis of ZnO films by ALD

ZnO films with different thicknesses, 40, 80, 120, and 180 nm were deposited on Si(100) substrates by ALD using a Savannah 100 ALD system from Cambridge Nanotech. Diethylzinc (DEZn) was used as the precursor for zinc and deionized water was used as the oxidation source. The growth cycle consists of precursor exposures and N_2_(99.9999%) purge following the sequence of DEZn/N_2_/H_2_O/N_2 _with corresponding duration of 0.1:5:0.1:5 s. After each N_2 _purging, the reactor was pumped down to 0.1 Torr. DEZn and H_2_O were fed into the chamber through separate inlet lines and nozzles. In the ALD method, reagents (precursors) are introduced sequentially into the growth chamber and when precursors reach the substrate, they are interspersed by cycles of purging with inert gas (N_2_). The opening and closing sequences of the valves were controlled by a computer. Precursor introduction was done by opening the inlet valve between the reservoir and reactor chamber while the outlet valve was closed. The pressures of the DEZn and H_2_O in the reactor chamber were approximately 1 and 2 Torr, respectively, monitored by a vacuum gauge. The substrate temperature was maintained at 177°C during the deposition. The reaction was repeated 400, 800, 1,200, and 1,800 cycles to obtain the ZnO films with different thicknesses and crystallographic features.

### Growth of ZnO nanorods through hydrothermal process

In this process, Zn(NO_3_)_2_ (ZNT) and hexamethylenetetramine (HMT) purchased from Sigma-Aldrich (St. Louis, MO, USA) were used as reagents. The ZnO nanorods were grown in aqueous solutions of zinc nitrate (Zn(NO_3_)_2_.6H_2_O) 0.01 M and hexamine ((CH_2_)_6_N_4_) in deionized water; the ZNT/HMT molar ratio was always {1:1}. The ALD-ZnO films were placed in face-up position into glass reactor with screw cap and then equal amounts of both ZNT and HMT solutions were added. The reactor was immersed in a water bath at 90°C with mild agitation during 4 h. Finally the samples were rinsed with deionized water for several times and dried at 90°C for several hours before characterization. The samples were structurally and morphologically characterized by X-ray diffraction (XRD) using a Philips X'Pert PW3040 diffractometer (PANalytical, Almelo, the Netherlands) with Cu-Kα radiation and field emission scanning electron microscopy in a Hitachi S-5500 Field Emission Gun (Hitachi Co., Tokyo, Japan) ultrahigh-resolution scanning electron microscope (FE-SEM) (0.4 nm at 30 kV) with a BF/DF Duo-STEM detector. Additionally, the composition was determined by energy dispersive X-ray spectroscopy (EDS) with an INCA-Energy EDS (Oxford Instruments, Oxfordshire, UK) attached to the FE-SEM; and the seed-textured ZnO layer surface was analyzed by atomic force microscopy (AFM).

## Results and discussion

### ZnO films by ALD

**Figure 1:**
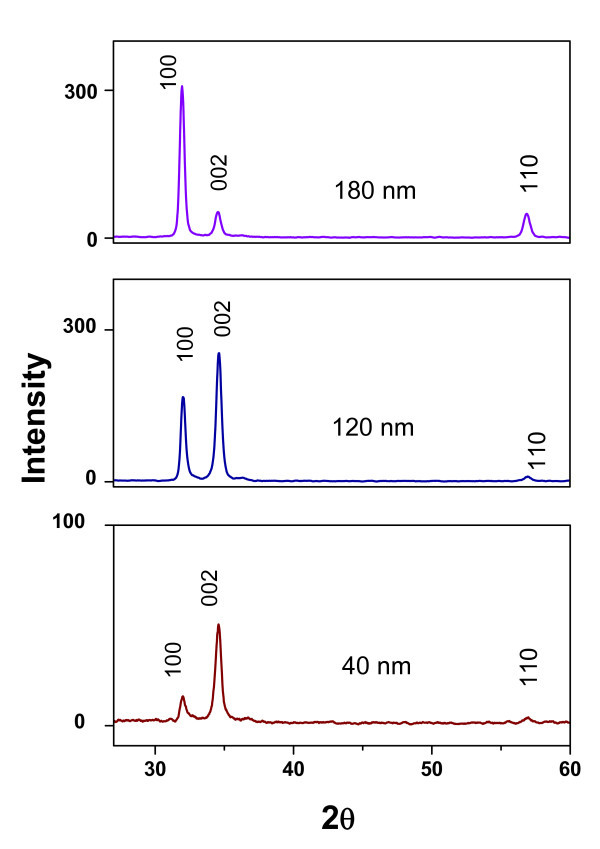
**X-ray patterns of ZnO films**. ZnO films with thicknesses between 40 and 180 nm.

XRD was performed on both substrates before and after nanorod growth. The crystallinity of the grown ZnO films obtained by ALD is shown in a typical XRD pattern in Figure 1. The X-ray spectra show well-defined Bragg peaks for the ZnO films corresponding to the planes (100), (002), and (110); these also confirm the wurtzite crystal structure of the whole set of samples (wurtzite structure, *a *= 3.249 Å and *c *= 5.201 Å) which is consistent with data of ZnO JCPDS no. 36-1451. All films were polycrystalline and at room temperature the strong signal centered at 34.5 indicates preferential growth in the (002) direction because the *c*-plane perpendicular to a substrate is the most densely packed and thermodynamically favorable plane in the wurtzite structure. This crystallographic condition induces some kind of *c*-axis texturing which depends of thickness. The degree of the orientation as function of thickness can be illustrated by the relative texture coefficient, which is given by Eq. 1:

(1) $$\text{T}{\text{C}}_{00\text{2}}=\left(I_{00\text{2}}∕{I_{00\text{2}}}^{0}\right)∕\left(I_{00\text{2}}∕{I_{00\text{2}}}^{0}+I_{\text{1}00}∕{I_{\text{1}}}_{00}^{0}\right)$$

where TC_002 _is the relative texture coefficient of diffraction peaks (002) over (100), *I*_002 _and *I*_100 _are the measured diffraction intensities due to (002) and (100) planes, respectively, and *I*_002_^0 ^and *I*_100_^0 ^are the corresponding values of standard PDF(36- 1451) measured from randomly oriented powder samples, so on this basis one can say that for materials with random crystallographic orientations, e.g., powders, the texture coefficient is 0.5. Now, about those ALD-ZnO films in which the highest peak was (002), as occurs in 40 and 120 nm films, the corresponding TC_002 _was increased as a confirming evidence of a preferential growth in that direction. The texture coefficient was 0.81, 0.60, and 0.14 for 40, 120, and 180 nm, respectively. It is also observed that preferential growth is disrupted with the increase of thickness given that the (100) peak at 31.7 becomes more intense for 180-nm films; it has been considered that the < 100 > orientation is favored due to the atomic disorder promoted with the ALD growth time. Texturing is apparently dependent of growth time because at longer times a crystallographic disorder is developed which limit the *c*-axis-oriented seeds and the crystal domain size. High texture in < 001 > direction will determine the quality of alignment and seed size the diameter of nanorods.

**Figure 2:**
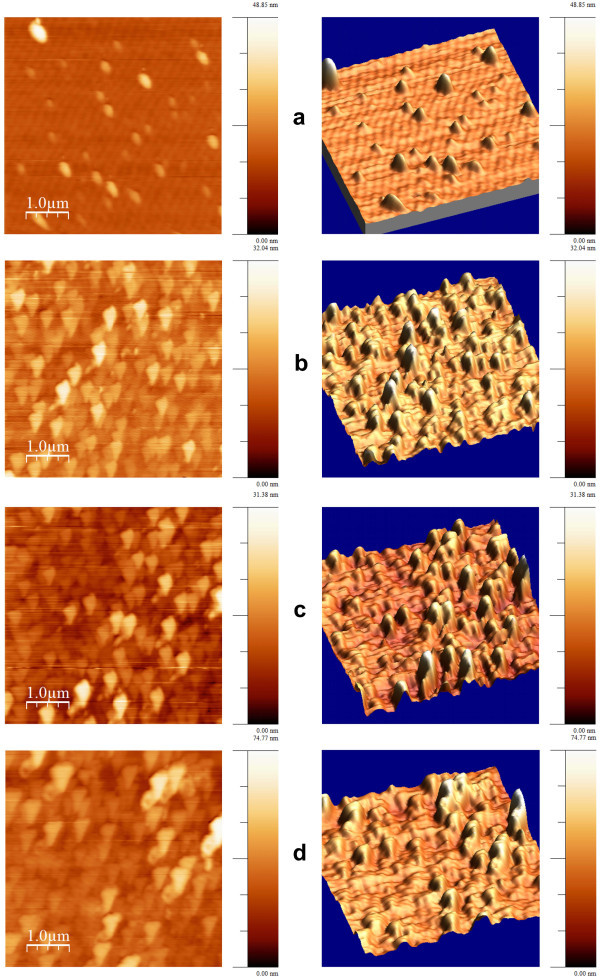
**AFM images**. ZnO films with different thicknesses: **(a) **40 nm, **(b) **80 nm, **(c) **120 nm, and **(d) **180 nm.

AFM images of ALD-ZnO films grown with different thicknesses are shown in Figure 2 to distinguish typical surface features previous to the hydrothermal process. These micrographs depict that with the thickness increasing, their roughness and surface defects also increase, thus allowing the formation of nucleation sites for ZnO nanorods growth. The ZnO films are composed of fine small grains (seeds) and these have average height (AH) that depends on the film thickness, if the ALD-ZnO films of 40 and 120 nm are observed one can see AH values of 18.2 and 31.4 nm, respectively. The differences in crystallographic and microstructural properties are significantly influenced by the ALD parameters such as the process time and flow rate. The increase of roughness could influence the ZnO nanorod growth due to the fact that surface defects augment acting as a barrier to nucleation sites. It must be a competence between the number of nucleation sites and the crystallographic orientation disrupted by surface defects formed at the ALD-ZnO film. Table 1 shows the measurements developed through the AFM images as shown in Figure 2; here, it is evident that a long-term ALD deposit leads to create higher surface defects that must have an influence for the nanorod growth as it is demonstrated by scanning electron microscopy (SEM) analysis. For films with thickness of 40, 80, 120, and 180 nm the roughness was 3.2, 5.5, 8.1, and 12 nm, respectively. From these results, it is evident that surface roughness is greater when the film thickness increases. Maximums at the surface are high-energy sites where nanorod nucleation will be privileged while depression sites could be the non-growth regions due to the absence of oriented seeds that favors ZnO nanorod growth.

**Table 1:** AFM features (roughness and height of textured ALD-ZnO films)

Cycles	Mean roughness (nm)	Mean height (nm)
400	3.2	18.18
800	5.5	19.25
1,200	8.1	20.73
1,800	12	31.41

**Figure 3:**
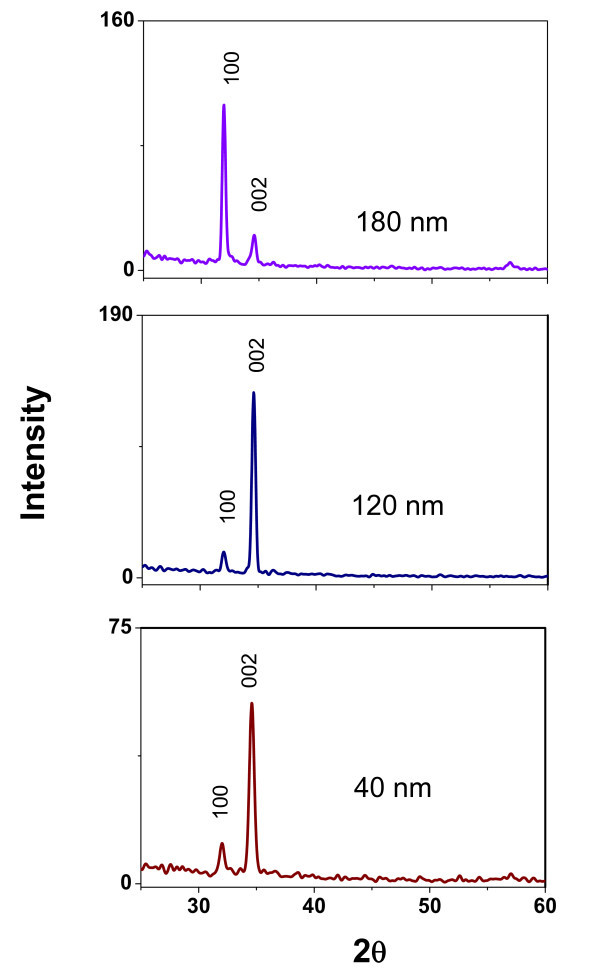
**X-ray patterns of ZnO nanorods**. ZnO nanorods grown on ALD-ZnO films with thicknesses between 40 and 180 nm.

After the nanorod growth on ALD-ZnO films with different textures, X-ray spectra were also recorded as depicted in Figure 3. XRD patterns of the resulting nanorod growth demonstrate that the orientation of the seed-textured ZnO films directly determines the orientation of the nanorods grown on these films.

From spectra, it is evident that the order of importance in intensity is maintained but the intensity ratio is changed as function of the nanorods growth type. In those ALD-ZnO films, in which the highest peak was (002) as occurs in 40, 80, and 120-nm films, the texture coefficient TC_002 _was increased as a confirming evidence of a preferential growth in that direction. The results indicate that the ZnO nanorod arrays are highly aligned on Si(100) substrate with *c*-axial growth direction, in addition, the diffraction intensity of the (002) peak surpasses others, which illustrates the *c*-oriented nature of the grown array. Otherwise, the TC_002 _of samples grown on textured ZnO films for 40, 120, and 180 nm is 0.84, 0.9, and 0.16, respectively; therefore the XRD results suggest that our samples are wurtzite ZnO nanorods with preferential *c*-orientation as confirmed by SEM analysis.

**Figure 4:**
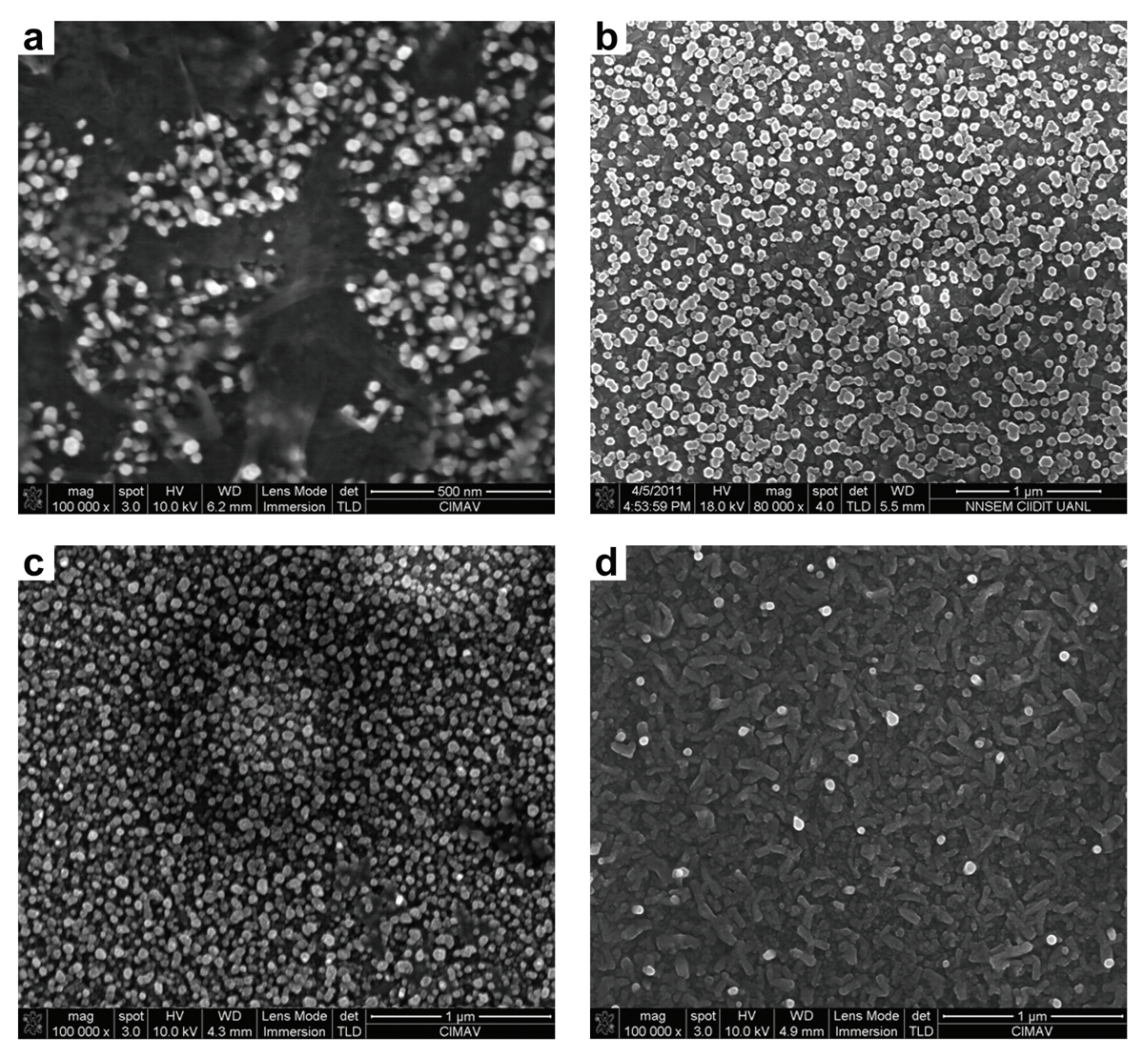
**Top view SEM images**. Images of ZnO nanorods grown on ALD-ZnO films: **(a) **40 nm, **(b) **80 nm, **(c) **120 nm, and **(d) **180 nm.

**Figure 5:**
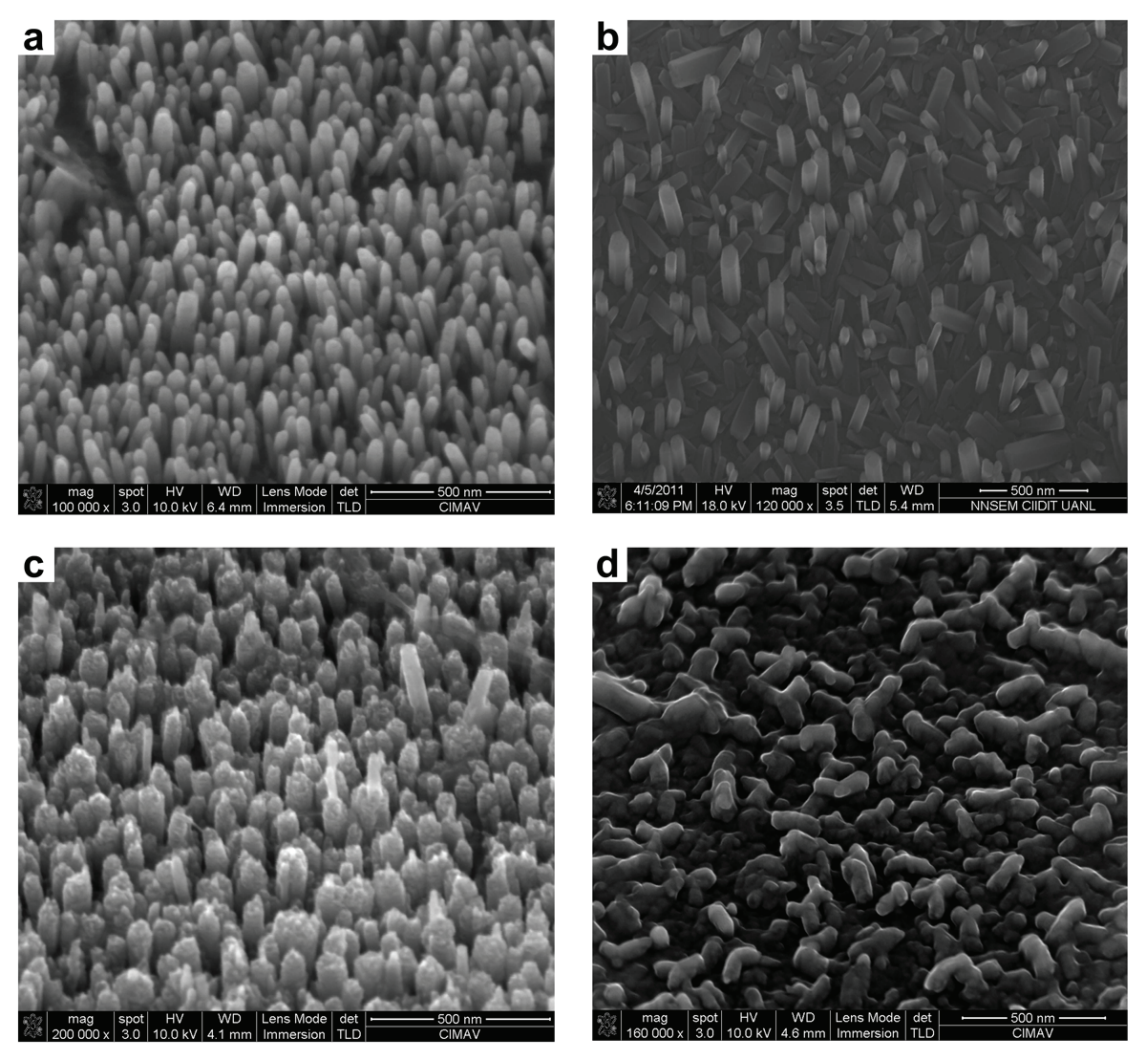
**Tilted SEM images**. Tilted images of ZnO nanorods grown on ALD-ZnO films of **(a) **40 nm, **(b) **80 nm, **(c) **120 nm, and **(d) **180 nm grown at 90°C, 4 h.

Figure 4 shows SEM images of the ZnO nanorod array grown by hydrothermal process on ALD-ZnO films with different thicknesses. The SEM images show a top view of the material deposited on the seed layer. It can be seen that density of ZnO nanorods depends on film thickness, whereas low density is typical from 40-nm films in Figure 4a, high density is present when a 120-nm film is used as seed layer in Figure 4b. Apparently thicknesses below 120 nm related with short ALD deposits give seeded surfaces with highly *c*-axis-oriented seeds whose size determines the nanorod diameter. Small thickness leads to small seed domain and thus, small diameters and low density of nanorods while long-term ALD experiments disrupt the ordered growth. The best conditions occur for middle-term ALD deposits in which the *c*-axis orientation is preserved and size domain increases to get larger diameters. The length of nanorods seems to be more dependent of hydrothermal process duration. The SEM images were also recorded in cross-section view to determine length and thickness for nanorods as shown in Figure 5. The measure of the nanorods size, population, thickness, and length was randomly chosen and obtained data were represented to obtain mean values. Therefore, the average nanorod size was fitted.

The nanorods have a narrow size distribution centered at about 34.5 ± 3.9 nm in diameter for the 40-nm films and 51.5 ± 5.2 nm for the 120-nm films. Cross-section view in Figure 5 demonstrated that the ZnO nanorods grew vertically with a mean length about 75.7 ± 14.3 nm for the 40 nm-films and 344.1 ± 97.6 nm for the 120-nm films. These geometric parameters are tunable to varying degrees by changing the growth time, ZNT concentration, or crystallography of seed-textured films. These results implied that our method is applicable to mass production of well-aligned ZnO nanorod arrays. All these results confirm that the hybrid method proposed to support nanorods is effective due to their high uniform distribution far and wide of the conducting substrate surface. The combined XRD and SEM data strongly suggest that *c*-axis texturing occurs across the ALD-ZnO film.

**Figure 6:**
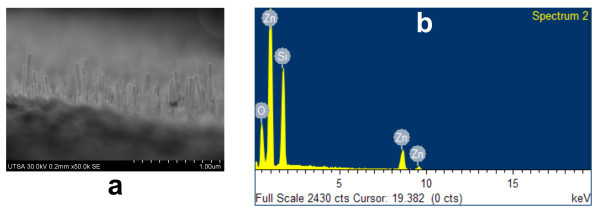
**Tilted SEM image and EDS spectra**. **(a) **Tilted image for ZnO nanorods grown on ALD-ZnO films of 80 nm and **(b) **EDS spectra to state the chemical nature of grown nanorods.

A tilted SEM image of ZnO nanorods grown on an 80-nm ALD-ZnO film is presented in Figure 6a to confirm that nanorod growth also occurs at thicknesses within the 40 to 120 nm range. On the other hand, Figure 6b shows the chemical composition of the nanorods determined by EDS. Only oxygen, zinc, and silicon are detected to confirm that the ZnO nanorods are the only phase present.

### Chemical reaction and growth mechanism

As stated by other authors, it is considered that the following reactions are involved in the crystal growth of ZnO nanorods [23-28].

(2) $${\text{C}}_{6}{\text{H}}_{12}{\text{N}}_{4}+6{\text{H}}_{2}\text{O}\hat{U}6\text{C}{\text{H}}_{2}\text{O}+4\text{N}{\text{H}}_{3}$$

(3) $${\left(\text{C}{\text{H}}_{2}\right)}_{6}{\text{N}}_{4}+\text{Z}{\text{n}}^{2+}®;{\left[\text{Zn}{\left(\text{C}{\text{H}}_{2}\right)}_{6}{\text{N}}_{4}\right]}^{2+}$$

(4) $$\text{N}{\text{H}}_{\text{3}}+{\text{H}}_{\text{2}}\text{O}Û\text{N}{{\text{H}}_{4}}^{+}+\text{O}{\text{H}}^{-}$$

(5) $$\text{Z}{\text{n}}^{2+}+4\text{N}{\text{H}}_{3}®;\text{Zn}{\left(\text{N}{\text{H}}_{3}\right)}_{4}^{2+}$$

(6) $$\text{Z}{\text{n}}^{2+}+4\text{O}{\text{H}}^{-}®;\text{Zn}{{\left(\text{OH}\right)}_{4}}^{2-}$$

(7) $$\text{Zn}{{\left(\text{N}{\text{H}}_{3}\right)}_{4}}^{2+}+2\text{O}{\text{H}}^{-}®;\text{ZnO}+4\text{N}{\text{H}}_{3}+{\text{H}}_{2}\text{O}$$

(8) $$\text{Zn}{{\left(\text{OH}\right)}_{\text{4}}}^{\text{2}-}®;\text{ZnO}+{\text{H}}_{\text{2}}\text{O}+\text{2O}{\text{H}}^{-}$$

(9) $${\left[\text{Zn}{\left(\text{C}{\text{H}}_{2}\right)}_{6}{\text{N}}_{4}\right]}^{2+}+2\text{O}{\text{H}}^{-}®;\text{ZnO}+{\text{H}}_{2}\text{O}+{\left(\text{C}{\text{H}}_{2}\right)}_{6}{\text{N}}_{4}$$

(CH_2_)_6_N_4 _is disintegrated into formaldehyde (CH_2_O) and ammonia (NH_3_) as shown in Eq. 2. Ammonia tends to disintegrate water to produce OH^- ^anions as described in Eq. 4. Finally, OH^- ^anions react with zinc (II) cations to form Zn(OH)_4_^2- ^(Eq. 6). In the growth process of ZnO nanorods, the concentration of OH^- ^anions is the dominant factor. Therefore, (CH_2_)_6_N_4 _that supplies OH^- ^anions plays an important key role in the growth of ZnO nanorods. Under the given pH and temperature, zinc (II) is thought to exist primarily as Zn(NH_3_)_4_^2+ ^and Zn(OH)_4_^2-^. The ZnO is formed by the dehydration of these intermediates. The solution method used a closed system that contains limited amounts of precursor. Along with the heterogeneous nanorod growth on the ZnO seed layer, there is also homogeneous nucleation of ZnO crystals in solution. This homogeneous nucleation consumes ZnO precursors rapidly and causes early termination of growth on the substrate. Therefore, depletion of the precursor is inevitable and growth rate decreases as reaction time increases.

**Figure 7:**
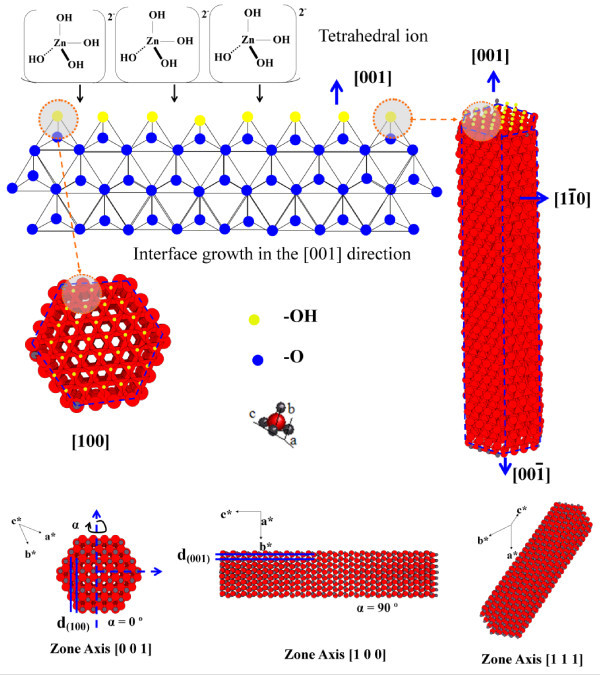
**Growth mechanism**. Proposed mechanism for ZnO nanorods growth at [001] direction.

**Figure 8:**
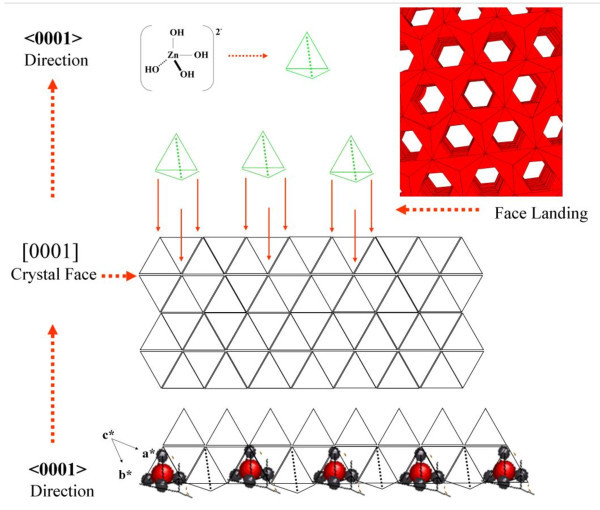
**Growth process of ZnO nanorods in the direction [001]**. The growth process is facilitated by the tetrahedral structure of the species Zn[(OH)_4_]^2- ^which fits well with the (001) surface polyhedra, this phenomenon (spatial resonance) increases the growth in this direction more than in another faces.

The reason for the *c*-axis-aligned nanorods is now examined. The microscopic details of seed formation have not been sufficiently understood and clarified to pinpoint which mechanism is responsible for the nanorod alignment. Some facts related with mechanisms at high temperatures, electrostatic processes, and electrical stability achieved by an exchange of charge mediated by surface states have been recently reported [26]. However, an explanation can be proposed in terms of our textured ALD-ZnO films. Textured ZnO films provide a surface formed mainly by seeds with *c*-axis-preferred orientation; these exposed basal planes of hexagonal rods are polar and have relatively high surface energy. As a result, the polar top planes attract more ion species promoting a faster growth rate and with this, the vertical-aligned ZnO nanorods emerge from the substrate. With all the mentioned before, it is reasonable to expect that ZnO nanorods orientation is determined by the nucleation and growth of the first few layers of zinc and oxygen atoms at the ALD seed layer through the fastest growth direction. This occurs because the polar {001} faces of wurtzite ZnO are electrostatically unstable and cannot exist without a mechanism to redistribute their surface charge and lower their free energy. According to reported models, optimized {001} surfaces have roughly 60% higher cleavage energy than the nonpolar {100} and {110} faces. Polar surfaces are generally stabilized by surface reconstruction or faceting; transfer of charge between surfaces or surface nonstoichiometry, including the neutralization of surface charge by adsorbed molecules. The following could enable the *c*-axis aligned nanorods: (1) Molecules present under the hydrothermal conditions adsorb onto nascent {001} surfaces and stabilize them relative to competing facets. In the decomposition of zinc nitrate to ZnO, these adsorbates would be primarily hydroxyl groups. The growth is favored due to the preference space of the reacting species, as illustrated in Figures 7 and 8. This shows the structure for the face (001), dots above of the polyhedral structure correspond to OH surface groups. The growth process is facilitated by the tetrahedral structure of the species Zn[(OH)_4_]^2- ^which fits well with the (001) polyhedral surface, this spatial resonance increases the growth in this direction more that in another faces. (2) The {001} surface energy depends on the crystal thickness so that very thin ZnO crystals prefer a {001} orientation, which is then kinetically locked-in as growth proceeds. (3) The first few atomic layers of ZnO must adopt a low-energy configuration different from the bulk lattice and later convert to the (001) orientation by a minor structural transformation. Notwithstanding all mentioned above, it is deemed that microscopic analysis of seed formation must be developed to pinpoint the right mechanism responsible for nanorod alignment.

## Conclusions

A simple seeding method for producing vertical ZnO nanorod arrays on Si(100) substrates is presented. By forming layers of textured ZnO films by ALD on a substrate, a seeded surface can be used to fabricate high-density vertical nanorod arrays. From the results, it is observed that thickness influences the texture of ALD-ZnO layer and thus, the crystallographic nature of the seed layer that determines the ulterior nanostructure growth type. Whereas short-term ALD deposit leads to create a surface with mostly *c*-axis-oriented seeds that favor the alignment, a long-term ALD deposit leads to create higher surface defects with polycrystalline seeds that promote disorder for ZnO nanorod growth. It is known that geometric parameters are tunable by changing the growth time and solution composition, with regards to the results of this work; this is also possible through changing seed density by controlling texture. Small thicknesses related with a short ALD deposit give seeded surfaces with small highly *c*-axis-oriented domains that promote small diameters with low density of nanorods while long-term ALD experiments disrupt the ordered growth. The best conditions occur for middle-term ALD deposits in which the *c*-axis orientation is preserved and size domain increases to get bigger diameters for nanorods. The arrays grown from aqueous solution feature a nanorod diameter, length, density, and orientation that make them highly suitable as the inorganic scaffold in efficient nanorod-polymer solar cells.

## Abbreviations

1D: one-dimensional; AFM: atomic force microscopy; AH: average height; ALD: atomic layer deposition; DEZn: diethylzinc; DSSCs: dye-sensitized solar cells; EDS: energy dispersed x-ray spectroscopy; FE-SEM: field emission scanning electron microscope; HMT: hexamethylenetetramine; JCPDS: Joint Committee on Powder Diffraction Standards; PDF: powder diffraction file; SEM: scanning electron microscopy; XRD: X-ray diffraction; ZNT: Zn(NO_3_)_2_.

## Competing interests

The authors declare that they have no competing interests.

## Authors' contributions

FP carried out the hydrothermal synthesis and drafted the manuscript. EM carried out the ALD-ZnO textured substrates, developed the XRD, AFM, and SEM studies and drafted the manuscript. MFM participated in discussion of results contributing with his experience on this topic and contributed with the writing of manuscript. EP contributed with fruitful discussions to the presented research. All authors read and approved the final manuscript.
